# Effect of inulin supplementation on clinical symptoms, inflammatory and oxidative stress markers in women with migraine: study protocol for a randomized clinical trial

**DOI:** 10.1186/s13063-023-07765-4

**Published:** 2023-11-11

**Authors:** Mahdi Vajdi, Fariborz Khorvash, Mohammad Hossein Rouhani, Abed Ghavami, Cain C. T. Clark, Gholamreza Askari

**Affiliations:** 1 Department of Community Nutrition, School of Nutrition and Food Science, Isfahan University of Medical Sciences, Isfahan, Iran; 2 Isfahan Neurosciences Research Center, Isfahan University of Medical Sciences, Isfahan, Iran; 3 Food Security Research Center and Department of Community Nutrition, School of Nutrition and Food Science, Isfahan University of Medical Sciences, Isfahan, Iran; 4 Institute for Health and Wellbeing, Coventry University, Coventry, CV1 5FB UK

**Keywords:** Migraine, Inulin, Supplementation, Inflammation, Oxidative stress

## Abstract

**Background:**

Migraine is a complex, chronic, and debilitating multifactorial disorder characterized by recurrent episodes of headache and related symptoms. It typically begins in early ages and is more prevalent in women than in men. Recently, the gut–brain axis has emerged as a new candidate that may be linked to neurological diseases. We hypothesize that selective modulation of the intestinal microbiota, oxidative stress, and inflammation through inulin supplementation may improve clinical outcomes in these patients. Therefore, this study aims to examine the effects of high-performance inulin supplementation on clinical symptoms, mental health, quality of life (QOL), intestinal permeability, and inflammatory and oxidative stress factors in women with migraine.

**Methods:**

This is a randomized, double-blind, placebo-controlled clinical trial involving 80 women with migraine who meet the inclusion criteria (aged between 20 and 50 years with a diagnosis of migraine by a neurologist based on the ICDH-3). Participants will be assigned to receive a daily dose of 10 g of inulin for 12 weeks (intervention group, *n* = 40) or 10 g of maltodextrin as a placebo for the same duration (control group, *n* = 40). The primary outcome will measure the variations in the frequency of headache experienced by the patients. Secondary outcomes will encompass serum levels of zonulin, high-sensitive C-reactive protein, total antioxidant capacity, total oxidant status, nitric oxide, mental status, QOL, duration, and severity of migraine attacks.

**Discussion:**

This clinical trial aims to evaluate the effect of inulin supplementation on inflammatory status, oxidative stress, intestinal permeability, clinical symptoms, mental health, and QOL in women with migraine. The findings of this trial could contribute to the identification of mechanistic action and evidence-based clinical guidelines that address gut microbiota manipulation to maximize health benefits in the management of clinical outcomes in migraine patients.

**Trial registration:**

Iranian Registry of Clinical Trials (www.irct.ir) (ID: IRCT20121216011763N58). Registration date: 23 April 2023.

**Trial status:**

The protocol is version 3.0, September 17, 2023. Recruitment began August 21, 2023, and is anticipated to be completed by March 22, 2024.

**Supplementary Information:**

The online version contains supplementary material available at 10.1186/s13063-023-07765-4.

## Introduction

### Background and rationale {6a}

Migraine is a complex, chronic, and debilitating multifactorial disorder, characterized by recurrent episodes of headache and related symptoms, such as photophobia, phonophobia, nausea, vomiting, vertigo, and cognitive dysfunction, which lasts for 4–72 h [1, 2]. The prevalence of migraine is estimated to be around 12% of the general population, with the highest occurrence observed among individuals aged 18 to 44 years. Women are more susceptible to migraines compared to men, with prevalence estimates ranging from 5 to 25% worldwide [3, 4]. Although the prevalence of migraine is generally lower in Asian populations compared to Western countries [5], it is noteworthy that the prevalence of this disease in Iran is approximately 15.1%, which is higher than global comparators [6].

The exact pathogenesis of migraine remains incompletely understood; however, there is a general consensus that it results from a complex interplay of vascular and neural events [7]. Several mechanisms have been proposed to explain the development of migraines, including neuro-inflammation, vascular dysfunction, and the gut–brain axis [7–10]. In recent years, there has been growing interest in the role of microbiota and gut health in the prevention and development of neurological disorders [11, 12]. The bidirectional interaction between the enteric and central nervous systems, known as the gut–brain axis, has been suggested to play a role in the association between the brain, gut microbiota, and cognitive function [13]. Furthermore, the composition of the gut microbiota plays a significant role in the gut–brain axis through direct connections with stimulating end terminals of the vagus nerve and indirect signaling involving inflammatory molecules, microbiota-derived neurotransmitters, and hormones [14]. Also in this case, the mechanism is bidirectional as the central nervous system (CNS) can modulate gut microbiota through the parasympathetic and sympathetic systems and by releasing neuroendocrine peptides [15]. Alterations in the profile of the intestinal microbiota may occur due to the influence of both physical and psychological stress factors. These stressors may result in dysbiosis, a condition where the composition of the gut microbiota undergoes alterations [16, 17].

Recent evidence suggests that oxidative stress and inflammation contribute significantly to the pathogenesis of migraines by triggering the abnormal activation of nociceptive sensory neurons [18, 19]. In this regard, migraine attacks may be neuro-protective responses to elevated oxidative stress and inflammation in the brain [20]. Moreover, it is important to note that a dysbiosis of the gastrointestinal tract microbiota and an increase in gut permeability could potentially trigger the activation of the hypothalamic–pituitary–adrenal axis. This activation is typically mediated by the release of pro-inflammatory cytokines such as (IL)-1β, high-sensitivity CRP (Hs-CRP), IL-6, tumor necrosis factor-alpha (TNF-α), and IL-8 [21, 22]. It is noteworthy that synthetic antioxidants such as 5-(5-substituted-1,3,4-oxadiazol-2-yl) benzene-1,2,3-triols and 5-substituted-1,3,4-thiadiazole-2-thiols have displayed very high and significant antioxidant activities in several studies [23, 24]. These findings raise the prospect of using oxidative stress modulator-synthetic antioxidants as therapeutic interventions for migraine patients.

Dietary components such as fiber, prebiotics, and probiotics have the potential to significantly influence the mechanisms by which the gut microbiota regulates immune function and gastrointestinal health [25]. Short-chain fatty acids (SCFAs), including butyrate, acetoacetate, and propionate, play a crucial role in maintaining gut barrier integrity [26]. In addition to their role in maintaining homeostasis and mitigating inflammation within the gastrointestinal tract, SCFAs are also known for their neuroprotective properties and their ability to modulate immune responses [26]. Interestingly, the supplementation of prebiotics, specifically fermentable fibers, to a high-fat diet has been reported to counteract the reduced levels of butyrate-producing bacteria and Bifidobacteria [27, 28].

The potential for supplementation with prebiotics and probiotics to modulate the frequency and severity of migraine attacks is an area of ongoing research. The exact mechanisms through which this modulation occurs remain somewhat elusive, but several hypotheses have been proposed. One such hypothesis suggests that prebiotics and probiotics may enhance the production of SCFAs in the gut. Another theory posits that these supplements could strengthen the integrity of the intestinal epithelium. Furthermore, these supplements may exert an anti-inflammatory effect by suppressing the nuclear factor kappa-B (NF-κB) pathway, thereby reducing levels of pro-inflammatory cytokines [29, 30]. However, these mechanisms necessitate further investigation to fully comprehend their implications in the context of migraine management. In 2019, a randomized clinical trial evaluated the effect of daily administration of a complex 14-strain probiotic mixture versus a placebo over 8 weeks in patients with chronic migraines and 10 weeks in those with episodic migraines. The administration of the probiotic mixture resulted in significant improvements in the frequency and severity of migraines, as well as a reduction in the usage of abortive medications within the studied population. Notably, these improvements were observed despite the absence of significant alterations in the serum levels of inflammatory biomarkers [29]. In another study conducted in Iran in 2021, a 12-week intervention with synbiotic led to a significant reduction in the average frequency of migraine attacks, the number of painkillers used, and gastrointestinal problems. Moreover, the synbiotic group exhibited lower levels of zonulin and Hs-CRP, which are markers of intestinal permeability and inflammation, respectively. However, the authors did not observe any beneficial effect of synbiotic supplementation on migraine severity and duration [10]. In a subsequent study, the administration of a probiotic mixture composed of seven bacterial strains resulted in approximately 25% reduction in the frequency of migraine attacks [31].

As previously discussed, functional foods such as probiotics and synbiotics have been shown to influence migraine symptoms in recent studies [10, 29, 32]. However, the role of prebiotics in this condition is still not well understood. Inulin-type fructans are a group of functional foods that can enhance the properties of beneficial intestinal bacteria. High-performance inulin (HP inulin) is a prebiotic with long-chain, high-molecular-weight mixes of inulin-type fructans. Inulin supplementation has been demonstrated to elicit several health benefits, including immune system regulation [33], anti-inflammatory effects [34], anti-depressant effects [35], and antioxidant effects [36]. Previous studies have indicated that prebiotics can induce specific changes in the activity and/or composition of the gut microbiota, particularly bifidogenic effects, which can have a beneficial impact on inflammation by balancing endothelial function and gut permeability [37, 38]. Therefore, prebiotic supplementation could be a beneficial strategy for reducing the frequency and severity of migraine attacks and improving the quality of life (QOL) for these patients. To the best of our knowledge, no published clinical trials have yet evaluated the impact of inulin on migraine patients. Consequently, this study is designed to investigate the potential effects of HP inulin supplementation on various aspects, including clinical symptoms, mental health, QOL, intestinal permeability, as well as inflammatory and oxidative stress markers in women suffering from migraines.

### Objectives {7}

The current study aims to evaluate the impact of HP inulin supplementation on inflammatory status, oxidative stress, intestinal permeability, clinical symptoms, mental health, and QOL in women with migraines. If this study confirms our hypothesis, inulin supplementation may be used to enhance the effects of existing migraine treatments.

### Trial design {8}

A randomized, parallel, two-arm, double-blind, and placebo-controlled superiority clinical trial will be conducted.

## Methods: participants, interventions, and outcomes

### Study setting {9}

This clinical trial will be carried out at the neurology clinic affiliated with Isfahan University of Medical Sciences in Isfahan, Iran.

### Eligibility criteria {10}

#### Inclusion criteria

The inclusion criteria will consist of patients who meet the following conditions:

- Aged between 20 and 50 years with a diagnosis of migraines by a neurologist (F.K), based on the International Classification of Headache Disorder-3 (ICDH-3)
- Residents of Isfahan city who are willing to participate in the study
- Fill out a written informed consent form prior to participation in this trial

#### Exclusion criteria

The exclusion criteria will consist of patients who meet the following conditions:

- Subjects who have/had other types of headaches such as tension-type headache or medication overuse headache
- Pregnant or breastfeeding subjects
- Subjects on a special diet or consuming nutritional supplements (probiotics, prebiotics, and dietary fiber supplements)
- Subjects who have/had gastrointestinal disorders like Crohn’s disease and ulcerative colitis, as well as other neurological disorders
- Subjects who have/had use of antibiotics and anti-acids drugs in three months past
- Subjects who are unwilling to continue with the study

### Reason for eliminating the data

Consumption of less than 80% of inulin supplements by the patient.

### Informed consent procedures 26a

Patients will be informed about the benefits and risks of the current trial. Subsequently, the principal investigator (M.V.) will explain the study’s purpose to the patients and obtain their written informed consent before they participate.

### Additional consent provisions for collection and use of participant data and biological specimens {26b}

The consent form provided to participants includes their agreement to use the data in future studies related to this one. However, they are not obligated to consent to any other studies beyond this.

### Interventions

#### Explanation for the choice of comparators {6b}

While receiving standard treatment, patients will be assigned to one of two groups: a treatment group receiving an inulin supplement or a control group receiving a placebo. The treatment group will receive one pack containing 10 g of inulin per day, while the control group will receive one placebo pack containing 10 g of maltodextrin per day for a duration of 12 weeks. Previous studies have shown that supplementation with 10 g of inulin per day is safe and does not cause any serious side effects [39, 40].

#### Intervention description {11a}

Upon recruitment into the trial, patients will be randomly assigned to one of two groups. The intervention group will receive one pack of 10 g inulin (Frutafit® TEX, Sensus; DP ≥ 23) daily for 12 weeks. The control group will receive one placebo pack containing 10 g maltodextrin (Zarfructose Company; dextrose equivalent [DE] = 14) daily. Maltodextrin was chosen as the placebo because it is easily digested and has a similar appearance and taste to inulin. Inulin and placebo will be packed with the same shape, color, weight, and size. A person who is unaware of the study details will label the packs containing inulin and maltodextrin as A or B. Patients will be instructed to continue their usual diet, physical activity, and medications until the intervention is completed. They will also be given instructions on how to use their supplements. Every 4 weeks, patients will receive packs of inulin and a placebo. Additionally, weekly follow-up phone calls and clinic visits will be used to evaluate side effects and treatment compliance.

#### Criteria for discontinuing or modifying allocated interventions {11b}

Patients may withdraw from the trial at any time for any reason. The investigator may withdraw patients from the trial to protect their safety or if they are unable or unwilling to continue with the trial. Furthermore, the reasons for each patient’s withdrawal from the trial will be explained in detail in the study results.

#### Strategies to improve adherence to interventions {11c}

Patients will be provided with inulin and placebo packs at weeks 1, 4, and 8. At the baseline, each phone call, and intermediate visits, all patients will receive instructions on how to use their supplements. They will be requested to return the packs with any remaining contents at the end of each 4-week period, and any side effects experienced will be recorded. Additionally, patients will be asked to complete a daily calendar immediately after consuming their assigned treatments. Adherence to intervention refers to the extent to which trial participants’ behavior aligns with their assigned intervention. Adherence to the assigned treatments will be assessed through the use of a daily calendar during each phone call and by counting the returned packs at the end of the trial. Compliance will be calculated using the following formula: Compliance rate = (packs taken / packs prescribed) × 100. A compliance rate below 80% will be classified as poor compliance.

#### Relevant concomitant care permitted or prohibited during the trial {11d}

Patients will receive standard care and treatment from a specialist doctor, which will not be altered during the study. In addition to their regular treatments, patients will only receive inulin supplements or a placebo. There are no restrictions on appropriate concurrent care for participants, who are also allowed to maintain their regular care.

### Provisions for post-trial care {30}

Standard treatments will continue after the trial concludes; however, placebo or inulin supplements will only be administered for a duration of 12 weeks during the trial.

### Outcomes {12}

This study will be conducted as a randomized double-blind placebo-controlled clinical trial to investigate the effects of inulin supplementation on clinical symptoms, inflammatory status, oxidative stress, intestinal permeability, mental health, and QOL in women with migraine. The primary outcome of this study is to evaluate the impact of inulin on the frequency of headaches in women diagnosed with migraines. In addition to this, the study will also focus on secondary outcomes, which include the assessment of serum levels of zonulin, Hs-CRP, total antioxidant capacity (TAC), total oxidant status (TOS), and NO. Furthermore, the mental health status, QOL, duration, and severity of migraine attacks will also be evaluated. The migraine symptoms, such as headache frequency, severity, and duration will be assessed by a neurologist. Participants will then be asked to complete questionnaires regarding the duration and frequency of the attacks during the intervention. The frequency of attacks will be recorded by the monthly incidence of headaches. The severity of pain will be assessed using a visual analogue scale (VAS) ranging from 0 to 10. Participants will be instructed to score 0 if they experience no pain and 10 if they suffer from agonizing pain. The study will measure the following parameters at the baseline and the end of the intervention: levels of zonulin, Hs-CRP, NO, TAC, and TOS, clinical symptoms, mental health status, and QOL. Blood samples will be collected from the patients both before and 12 weeks after the intervention with either inulin or placebo. These samples will be drawn in the morning (8–10 am) under fasting conditions. Following collection, serum will be separated from the blood samples through centrifugation and then aliquoted into 0.5-ml microtubes. The serum samples will subsequently be stored at − 70 °C in a freezer until further analysis.

### Participant timeline {13}

**Fig. 1:**
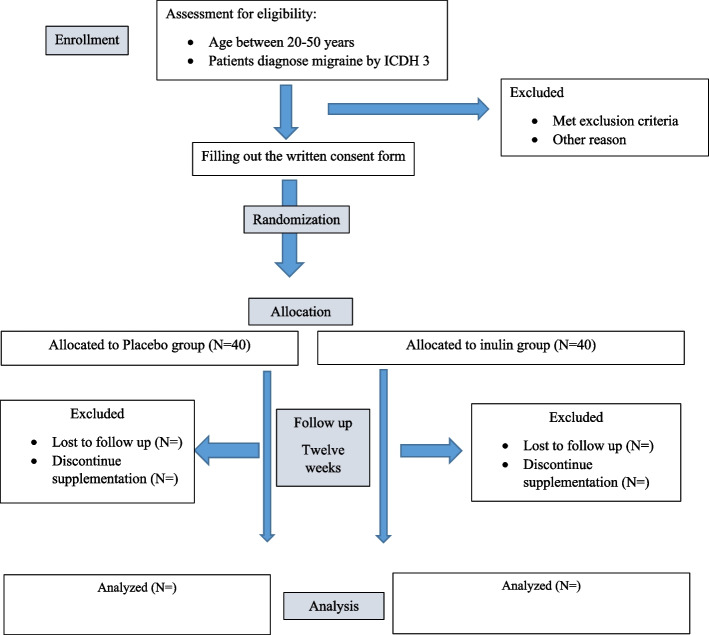
The protocol flow chart and timeline of the study

The protocol flow chart and timeline of the study are illustrated in Fig. 1 and Table 1, respectively. The study protocol was designed and developed in accordance with the Standard Protocol Items: Recommendations for Interventional Trials (SPIRIT) 2013 checklist (Additional file 1, SPIRIT Checklist).

**Table 1:** Schedule of enrollment, intervention, and assessment based on SPIRIT guidelines

		Registration	Allocation	Post-Allocation												Close-out
Time point (week)		-W_1_	W_0_	W_1_	W_2_	W_3_	W_4_	W_5_	W_6_	W_7_	W_8_	W_9_	W_10_	W_11_	W_12_	W_13 (starting)_
Registration	Eligibility testing	*														
	Informed consent	*														
	Randomization		*													
	Allocation		*													
	Patient instruction			*												
Intervention	Supplementation			*	*	*	*	*	*	*	*	*	*	*	*	
	Compliance			*	*	*	*	*	*	*	*	*	*	*	*	
	Adverse events			*	*	*	*	*	*	*	*	*	*	*	*	
Assessment	Demographic			*												*
	Anthropometric indices			*												*
	Physical activity			*												*
	Dietary intake			*												*
	Mental status			*												*
	Quality of life			*												*
	Clinical features			*												*
	Biochemical assays			*												*
	Supplement checklist															*

Demographic variable including: type of migraine (episodic and chronic), age, anthropometric indices, education status (university graduated, and non-university graduated), marital status (single and married), migraine in first relatives, gastrointestinal problems (bloating, constipation and diarrhea), medication (beta-blockers, anticonvulsant drugs, Tricyclic Antidepressants (TCA), Tetracyclic Antidepressant (TeCA), Serotonin-Norepinephrine Reuptake Inhibitor (SNRI) or mixture of them); Anthropometric indices including: body weight (BW), body mass index (BMI) and waist circumference (WC); Mental including: depression, anxiety, stress; Quality of life including: migraine Specific Quality of Life (MSQ) and the headache impact test-6 (HIT-6); Clinical features including: frequency, severity, duration; Biochemical assays including: serum zonulin, High-sensitive C reactive protein (Hs-CRP), total antioxidant capacity (TAC), total oxidant status (TOS), nitric oxide (NO)

### Sample size {14}

The sample size for the present study was calculated based on means and standard deviations for the frequency of attacks, as the main outcome of the study of Ghavami et al. [41], by considering a 95% confidence interval and 80% power (*α* = 0.05 and *β* = 0.2). Frequency of attacks was also considered a main outcome, and based on a previous study, the sample size was calculated as 40 patients for each group. All patients who meet the inclusion criteria and are willing to participate in the trial will be included until the estimated sample size is completed.

### Recruitment {15}

The participant composition will consist of 80 patients with migraine recruited from a neurology clinic affiliated with Isfahan University of Medical Sciences in Isfahan, Iran. Our recruitment methods include in-person visits and written materials (such as flyers and brochures). We direct all interested and potential volunteers to a neurologist who will check their eligibility criteria. We obtain written informed consent from all eligible participants who voluntarily join the study. Patients who meet the ICDH-3 criteria [42] for diagnosing migraine will be assessed for eligibility criteria. An expert neurologist (F.K.) will then differentiate migraine from other types of headaches such as tension-type headache (TTH), trigeminal autonomic cephalagia (TACs), medication overuse headache (MOH), cluster headache (CH), and headache due to menstrual cycle.

### Migraine diagnosis

According to the ICHD-3, a neurologist will diagnose migraines. ICDH-3 is a well-known and up-to-date diagnostic instrument used in clinical research for headaches. Based on the ICDH-3 criteria, an individual must have at least five attacks that fulfill criteria A–C:

A) Headache attacks lasting 4–72 h (when untreated or unsuccessfully treated)
B) Headache with at least two of the following four characteristics:
  1. Unilateral location
  2. Pulsating quality
  3. Moderate or severe pain intensity
  4. Aggravation by or causing avoidance of routine physical activity (e.g., walking or climbing stairs).
C) During a headache, at least one of the following:
  1. Nausea and/or vomiting
  2. Photophobia and phonophobia

### Assignment of interventions: allocation

#### Sequence generation {16a}

The present study will be conducted as a randomized double-blind, placebo-controlled study using a stratified randomization method. Stratified permuted block randomization will be employed to balance age (20–35 and 35–50 years) between the two groups over time. Within each of the aforementioned strata, eligible patients will be randomly assigned in a 1:1 ratio to receive either inulin or placebo with a block size of four. A block size of four will be constructed in six different arrangements (blocks) which will be created for each stratum to assign patients to the trial groups. The sequence of these blocks will be generated for each stratum using a random number generator available at randomizer.org. A person not involved in the study will randomly divide patients into two groups: intervention (inulin, *n* = 40) and control (placebo, *n* = 40).

#### Concealment mechanism {16b}

To ensure blinding, packs of inulin or placebo will be labeled as A and B by an independent person who is not involved in the trial. Both patients and investigators will be unaware of the treatment assignment. The allocation sequence will be concealed from those assigning patients to the intervention groups using opaque sealed envelopes. Randomization codes will only be unlocked after all participants have completed the trial protocol.

#### Implementation {16c}

An independent clinical research coordinator will assign patients to either the intervention or placebo group. The generation of the allocation sequence for patients will be monitored by a second researcher (MH.R).

### Assignment of interventions: blinding

#### People who will be blind {17a}

This study will be conducted as a double-blind study, meaning that both investigators and patients will be unaware of which group they are assigned to. Additionally, inulin and placebo will be packed with identical shape, color, weight, and size.

#### Procedure for emergency unblinding if needed {17b}

The labels on the supplements will only be revealed at the final analysis stage of the study. Any unblinding that occurs during the study will be recorded and reported in the Iranian Registry of Clinical Trials, the Ethics Committee of Isfahan University of Medical Sciences, and in the final results of the study.

### Data collection and management

#### Plans for assessment and collection of outcomes {18a}

##### Demographic variables

Demographic variables, including the type of migraine (episodic and chronic), age, anthropometric indices (height, body weight (BW), waist circumference (WC), and body mass index (BMI)), education status (university graduated and non-university graduated), marital status (single and married), migraine in first relatives, gastrointestinal problems (bloating, constipation, and diarrhea), medication (beta-blockers, anticonvulsant drugs, tricyclic antidepressants (TCA), tetracyclic antidepressant (TeCA), serotonin–norepinephrine reuptake inhibitor (SNRI)), and physical activity will be obtained from each participant at the initiation and end of the intervention using validated tools.

##### Migraine symptoms assessment

Clinical features of migraine at the initiation and end of the intervention were obtained from all participants. The migraine symptoms, such as headache frequency (number of attacks per month), severity, and duration (mean duration of headache attacks in a day per month) will be assessed by a neurologist. Participants will then be asked to complete questionnaires regarding the duration and frequency of the attacks during the intervention. The severity of pain will be assessed using a VAS ranging from 0 to 10. Participants will be instructed to score 0 if they experience no pain and 10 if they suffer from agonizing pain. The headache impact test-6 (HIT-6) scale will also be used to evaluate the degree of disability due to migraine at baseline and at the end of the trial. The HIT-6 questionnaire examines the patient’s disability as a score. The reliability and validity of this questionnaire have been established in an Iranian population [43]. Moreover, clinical symptoms of migraine, such as the migraine headache index score (MHIS), headache diary result (HDR), and migraine index (MI), will be calculated using the following formula:

$$\mathrm{MI }=\mathrm{ frequency }\times \mathrm{ severity}$$

$$\mathrm{HDR }=\mathrm{ frequency }\times \mathrm{ duration}$$

$$\mathrm{MHIS }=\mathrm{ frequency }\times \mathrm{ duration }\times \mathrm{ severity}$$

##### Dietary intake assessment

To evaluate dietary intake, participants will be asked to maintain a 3-day food record (one weekend and two weekdays). A trained nutritionist, who will be blinded to the treatment assignment, will instruct patients on how to complete their 3-day records. The amounts of each food item and drink will then be converted to grams per day using Iranian Household Measures [44] and subsequently converted to the value of nutrients and calories using Nutritionist 4 software (First Databank Inc., Hearst Corp., San Bruno, CA, 161 USA).

##### Physical activity assessment

PA status will be assessed via the International Physical Activity Questionnaire (IPAQ), a self-administered, 7-day recall instrument whose validity and reliability have been previously approved among the Iranian population [45]. PA levels will be reported as metabolic equivalent hours per day (MET/h/day).

##### Anthropometric assessments

BW will be measured without shoes and with minimal clothing using a Seca scale with an accuracy of 100 g. Height will be measured in a standing position without shoes using a portable stadiometer to the nearest 0.5 cm. Body mass index (BMI) will be calculated using the following formula: weight (kg)/height squared (m^2^). WC will be measured by a trained dietitian at the midpoint between the lower costal margin and the iliac crest using a tape measure to the nearest 0.1 cm. Hip circumference (HC) will be measured over the widest part of the buttocks and recorded to the nearest 0.1 cm. Waist-to-hip ratio (WHR) will be calculated as WC divided by HC.

##### Assessment of quality of life (QOL)

The impact of headache on QOL will be assessed using the validated Migraine Specific QOL (MSQ) questionnaire and the HIT-6. The HIT-6 is a validated questionnaire that contains six questions with five options: “never” (score = 6), “rarely” [8], “sometimes” [10], “very often” [11], and “always” [13]. The total possible score ranges from 36 to 78. Scores between 36 and 49, 50 and 55, 56 and 59, and ≥ 60 indicate that the headache has no, moderate, substantial, and severe impact on the quality of life, respectively [46, 47]. The MSQ is a valid and reliable questionnaire that measures QOL among migraine patients over the past 4 weeks. It consists of three scales assessing three QOL domains: (1) Role Restrictive (RR), which includes seven items that assess how patients’ performance of normal activities is limited by migraine; (2) Role Preventive (RP), which consists of four items that assess how patients’ performance of normal activities is interrupted by migraines; and (3) Emotion Function (EF), which consists of three items that assess the impact of migraine on the respondent’s emotions. The item responses range from 1 to 6, with “1” representing “None of the time”, “2” representing “A little bit of time”, “3” representing “Some of the time”, “4” representing “A good bit of the time”, “5” representing “Most of the time”, and “6” representing “All of the time”. The total possible score ranges from 14 (minimal summation) to 84 (maximum summation). These scores are reverse-coded and standardized to a 0–100 scale using the following formula: (total score of each subject − 14) / 70 × 100. Higher scale scores indicate better migraine-related QOL [48].

##### Assessment of mental health status

The Depression, Anxiety, Stress Score (DASS-21) questionnaire is a self-report questionnaire that comprises 21 items, with 7 items per subscale: depression, anxiety, and stress. Patients are required to score each item on a scale ranging from 0 (did not apply to me at all), 1 (applied to me to some degree), 2 (applied to me to a considerable degree), and 3 (applied to me very much). Sum scores are computed by adding up the scores on the items per (sub) scale and multiplying them by a factor of 2. Subjects will be classified to five levels in terms of depression, where the scores of 0–4, 5–6, 7–10, 11–13, and ≥ 14 indicate the normal, mild, moderate, severe, and extremely severe depression, respectively. Also, about anxiety, the score of 0–3, 4–5, 6–7, 8–9, and ≥ 10 indicate normal, mild, moderate, severe, and extremely severe anxiety, respectively. Stress is one of the other aspects of mental status and scores of 0–7, 8–9, 10–12, 13–16, and ≥ 17 in this questionnaire indicate normal, mild, moderate, severe, and extremely severe stress, respectively [49, 50].

### Plans to promote participant retention and complete follow-up {18b}

The potential benefits of inulin supplementation for preventing complications in patients with migraines will be discussed to encourage their full participation in the study. The research team will maintain regular contact with participants via telephone. Additionally, they will employ strategies to enhance participant retention, such as sending reminders for taking the supplementation and scheduling post-intervention visits.

### Data management {19}

One of the researchers will oversee the coding, security, and storage of data. Data will be entered electronically at the participating site, and the forms will be securely stored on file. Furthermore, they will double-check data entry and values. If any patient reports adverse events, additional information will be gathered to determine whether to exclude them from the study. According to the Medical Ethics Committee Criteria, unblinding is permissible in this situation, allowing us to specify whether the patient received inulin or a placebo.

### Confidentiality {27}

Each participant will be assigned a unique study identification code, which will be used to store research data. The research team will have access to the code list during the study, which will be documented and protected by the principal investigator according to research guidelines after the study concludes. Personal details of participants will not be disclosed in any publications.

### Plans for collection, laboratory evaluation, and storage of biological specimens for genetic or molecular analysis in this trial/future use {33}

Laboratory assessments will be conducted at the beginning and conclusion of the study. A 10-ml blood sample will be obtained after a 12-h fasting period and centrifuged for 10 min at a speed of 2500 rpm at room temperature. The serum samples will be divided into microtubes and promptly frozen at − 70 °C. The levels of zonulin and Hs-CRP in the serum will be measured using ELISA (ZellBio, Germany) and turbidimetric (BIOREX, Iran) assay kits, respectively. The serum levels of TOS, TAC, and NO status will be assessed using the Fox1, Cuprdc, and Griess methods, respectively, with the aid of commercial kits (Kiazist Life Sciences, Iran). The Oxidative Stress Index (OSI) will be calculated using the following formula: OSI = [(TOS, μmol/l)/ (TAC, μmol/l) × 100].

### Statistical methods

#### Statistical methods for primary and secondary outcomes {20a}

Statistical analyses will be conducted using SPSS (version 21.0) software. A *P*-value < 0.05 will be considered statistically significant. The one-sample Kolmogorov–Smirnov test will be used to evaluate the normality of the distribution of data. Regarding missing values, the data analysis will be subjected to intention-to-treat (ITT) analysis. We will employ the multiple imputation approach to perform ITT. In order to compare the baseline value of qualitative and quantitative variables, the chi-square test and *t*-test will be used. Categorical variables will be expressed as number and percentage, while quantitative data will be presented as mean ± standard deviation. Between-group differences will be evaluated using a Mann–Whitney *U* test and independent *t*-test. Differences within the group will also be assessed with a Wilcoxon rank-sum test and paired *t*-test. To control for confounders, an analysis of covariance (ANCOVA) will be carried out.

The primary results of this study include the evaluation of clinical symptoms of patients, such as headache frequency, duration, and severity. The secondary results encompass the evaluation of the serum levels of zonulin, Hs-CRP, TAC, TOS, NO, mental health status, and QOL by questionnaire.

#### Interim analyses {21b}

The recruitment of participants for the trial and their engagement in the intervention will be diligently monitored. The continuation criteria will be reviewed periodically throughout the course of the study. An intention-to-treat analysis will be conducted for participants who drop out during treatment sessions or follow-up assessments. It is important to note that participants who drop out will not be excluded from the data analysis. The study will be stopped upon the completion of participant enrollment, provided that no serious adverse events transpire during the course of the study. In the event that adverse events do occur, the study will be promptly discontinued. Adverse events encompass occurrences such as death, disability, and severe allergic reactions that have a substantial impact on the patient’s health. This trial does not include any planned interim analyses.

#### Methods for additional analyses (e.g., subgroup analyses) 20b

No subgroup analyses are planned.

#### Methods in analysis to handle protocol non-adherence and any statistical methods to handle missing data {20c}

The analysis will follow an ITT approach, which will include the data from the excluded patients in the results. The reason for each patient’s withdrawal, especially the occurrence of any side effects, will be explained in detail. Missing data will be addressed by multiple imputations.

#### Plans to give access to the full protocol, participant-level data, and statistical code {31c}

The corresponding author will provide the dataset if there are any logical requests for data in line with the present protocol. Otherwise, all information will be kept confidential.

### Oversight and monitoring

#### Composition of the coordinating center and trial steering committee {5d}

The researchers and all aspects of the study will be supervised by the Ethics Committee and the Vice-Chancellor of Isfahan University of Medical Sciences, who will ensure that the study adheres to ethical principles and protects the patient’s health and dignity. In case of any ethical violations, the study may be corrected or terminated. The principal investigator is the lead researcher and main coordinator of the trial. The principal investigator and research physician are in charge of recruiting, treating, and following up with participants. A steering committee will oversee the entire study process. The study team will also communicate regularly to monitor progress.

### Composition of the data monitoring committee, its role, and reporting structure {21a}

The academic committee of Isfahan University of Medical Sciences will conduct impartial and continuous data monitoring.

### Adverse event reporting and harms {22}

According to previous studies, supplementation with inulin at a dosage of 10 g per day has been found to be safe and devoid of serious side effects [39, 40]. In the event of any complications arising from inulin supplementation, the intervention will be promptly discontinued, and the necessary medical measures will be taken. Any potential adverse effects will be reported to the Ethical Committee of Isfahan University of Medical Sciences. Based on the severity and nature of the adverse effects associated with supplementation, the ethical committee will determine whether to exclude the patient from the study or assign an exclusive code or undertake other appropriate actions.

### Frequency and plans for auditing trial conduct {23}

This study will be conducted under the guidance of the Ethics Committee of Isfahan University of Medical Sciences. The Ethics Committee will oversee the quality, validity, and adherence to ethical standards by the researchers throughout the study period, with at least two monitoring sessions. A report will be submitted to the auditor every 3 months.

### Plans for communicating important protocol amendments to relevant parties (e.g., trial participants, ethical committees) {25}

Any changes to the protocol that may impact the implementation of the trial, such as modifications to study sample size, objectives, participants, or study procedures, must receive approval from the ethical committee of Isfahan University of Medical Sciences to be implemented. All changes will be reported in detail at https://irct.ir/.

### Dissemination plans {31a}

Final data and findings will be presented in official publications.

## Discussion

This study is, to the best of our knowledge, the first clinical trial evaluating the effects of inulin on migraine symptoms, mental health status, QOL, TOS, TAC, NO, zonulin, and Hs-CRP in women with migraines. Given the high prevalence of migraines and their impact on patients, it is imperative to explore novel and more effective therapeutic approaches for their treatment to alleviate pain and migraine attacks.

While the therapeutic effects of gut microbiota manipulation through the administration of probiotics/prebiotics have been discussed, there remains a dearth of information and unclear mechanisms regarding their potential to improve clinical outcomes in migraine patients. Moreover, the potential advantages of these biopharmaceuticals for individuals afflicted with migraines, who often endure headaches and a reduced quality of life, have not been extensively explored. This trial aims to address this critical research gap by investigating and comparing biomarkers of gut permeability, oxidative stress, and inflammation following inulin supplementation, as well as their subsequent impact on clinical outcomes among women with migraines. The innovative aspects of this clinical trial include (1) testing hypotheses related to inulin supplementation that involve a novel concept about the gut–brain axis, for which there is limited and incomplete data available in these subjects; (2) focusing on individuals at high risk for low quality of life, including those with migraines; and (3) conducting a 3-month follow-up with participants. Importantly, our findings could have a significant impact on clinical practice and public health by providing a simple and efficacious adjunctive approach for the prevention or amelioration of migraine headaches if our hypotheses are supported. The primary challenge of our study will be participant adhesion and retention. This challenge will be managed by the project coordinator who will have weekly contact with participants in which any concerns or issues will be immediately addressed. If these supplements are shown to be effective in the prevention or management of migraine headaches, they would represent a very attractive alternative or adjunctive therapy to current medications.

## Trial status

The protocol is version 3.0, September 17, 2023. Recruitment began August 21, 2023, and is anticipated to be completed by March 22, 2024.

### Supplementary Information

**Additional file 1.** SPIRIT 2013 Checklist: Recommended items to address in a clinical trial protocol and related documents*. 

## Data Availability

The first and corresponding authors will have access to all the results and make the final decision to terminate the trial. The non-identifiable individual patients’ data will be version 3.0, September 2023 and will be made available to other researchers in academic institutions.

## Abbreviations

ANCOVA: Analysis of covariance
BW: Body weight
BMI: Body mass index
CONSORT: Consolidated Standards of Reporting Trials
CH: Cluster headache
DASS-21: Depression, Anxiety, Stress Score
EF: Emotion Function
HIT-6: Headache impact test-6
Hs-CRP: High-sensitive C reactive protein
ICDH-3: International Classification of Headache Disorder-3
IRCT: Iranian Registry of Clinical trial
IPAQ: International Physical Activity Questionnaire
MIDAS: Migraine Disability Assessment Scale
MIHS: Migraine headache index score
MOH: Medication overuse headache
MSQ: Migraine Specific Quality of Life
NO: Nitric oxide
PA: Physical activity
RP: Role Preventive
RR: Role Restrictive
SPIRIT: Standard Protocol Items: Recommendations for Interventional Trials
SNRI: Serotonin–norepinephrine Reuptake Inhibitor
SD: Standard deviation
TTH: Tension-type headache
TCA: Tricyclic antidepressants
TeCA: Tetracyclic antidepressant
TAC: Total antioxidant capacity
TOS: Total oxidant status
TACs: Trigeminal autonomic cephalagia
VAS: Visual analog scale
WC: Waist circumference
